# Understanding the healthcare experiences and needs of African immigrants in the United States: a scoping review

**DOI:** 10.1186/s12889-019-8127-9

**Published:** 2020-01-08

**Authors:** Ogbonnaya I. Omenka, Dennis P. Watson, Hugh C. Hendrie

**Affiliations:** 1 College of Pharmacy and Health Sciences, Butler University, 4600 Sunset Avenue, PHSB 404E, Indianapolis, IN 46208 USA; 2 Center for Dissemination and Implementation Science, Department of Medicine, College of Medicine, University of Illinois at Chicago, Chicago, IL USA; 3 Department of Psychiatry, Indiana University School of Medicine, Indianapolis, IN USA

**Keywords:** Immigrant health, African immigrant, Scoping review, Health experience, Health and culture, Healthcare access, health disparities

## Abstract

**Background:**

Africans immigrants in the United States are the least-studied immigrant group, despite the research and policy efforts to address health disparities within immigrant communities. Although their healthcare experiences and needs are unique, they are often included in the “black” category, along with other phenotypically-similar groups. This process makes utilizing research data to make critical healthcare decisions specifically targeting African immigrants, difficult. The purpose of this Scoping Review was to examine extant information about African immigrant health in the U.S., in order to develop lines of inquiry using the identified knowledge-gaps.

**Methods:**

Literature published in the English language between 1980 and 2016 were reviewed in five stages: (1) identification of the question and (b) relevant studies, (c) screening, (d) data extraction and synthesis, and (e) results. Databases used included EBSCO, ProQuest, PubMed, and Google Scholar (hand-search). The articles were reviewed according to title and abstract, and studies deemed relevant were reviewed as full-text articles. Data was extracted from the selected articles using the inductive approach, which was based on the comprehensive reading and interpretive analysis of the organically emerging themes. Finally, the results from the selected articles were presented in a narrative format.

**Results:**

Culture, religion, and spirituality were identified as intertwined key contributors to the healthcare experiences of African immigrants. In addition, lack of culturally-competent healthcare, distrust, and complexity, of the U.S. health system, and the exorbitant cost of care, were identified as major healthcare access barriers.

**Conclusion:**

Knowledge about African immigrant health in the U.S. is scarce, with available literature mainly focusing on databases, which make it difficult to identify African immigrants. To our knowledge, this is the first Scoping Review pertaining to the healthcare experiences and needs of African immigrants in the U.S.

## Background

The health of African immigrants in the United States (U.S.) is a vastly under-studied topic, despite the rapidly increasing size of the population and its uniqueness. African immigrants make up about 5% of the U.S. population, which represents a 41% increase from the year 2000 [1]. More than 36% of them arrive from West Africa, followed by 29 and 17% from Eastern and Northern Africa, respectively. Over 14% of African immigrants in the U.S. come from Nigeria, followed by 10% from Ethiopia [2]. Factors contributing to the migration of Africans to the U.S. include family reunification, political disturbances in their country of origin, and education. Other reasons include the diversity lottery program, and brain drain [3, 4]. For instance, many African physicians and nurses migrate to the U.S. for higher-paying opportunities, leaving behind dilapidated health systems in their home countries [5, 6]. The healthcare experiences and needs of African immigrants are not universal, and research has shown there is considerable variation in healthcare experiences across populations [7, 8]. Prior to their arrival in the U.S., many African immigrants face severe health threats such as war, extreme poverty and mental health challenges, in their countries of origin [9]. Consequently, many African immigrants already carry significant health vulnerabilities upon arrival in the U.S., which can only worsen without proper healthcare access [10].

The paucity of knowledge regarding the healthcare experiences and needs of African immigrants in the U.S. due to two main factors: the absence of research or funding on immigrant health focused on this population [11], and the view that all black populations in the U.S. are the same [12]. The majority of research on immigrant healthcare in the U.S. has concentrated on populations from Latin and some Asian countries. However, the healthcare needs and experiences of other immigrant populations cannot be assumed to be identical to those of African immigrants. Also, African immigrants, which primarily comprises African Americans and Caribbean immigrants are often included in the “black” category [13–17]. This monolithic view of the black population in the U.S. bears serious health and healthcare implications for African immigrants, because while an Africa-born black immigrant and a U.S.-born black citizen may be phenotypically similar, their health beliefs and health outcomes may differ [18, 19]. For instance, babies born to Africa-born black mothers were found to have higher birth weights than those born to U.S.-born black mothers [20]. Also, African immigrants have shown lower prevalence of cardiovascular risk factors, including hypertension and diabetes, than African Americans [17]. Merging these groups obscures the distinctions that may exist within them, including the unique cultural backgrounds and healthcare experiences of African immigrant community [15–17, 21, 22].

Previously published studies have sought to gain insight into African immigrant health in the U.S., with focus ranging from barriers to healthcare—including cancer and HIV screenings [9, 10, 13, 23–25]—to dietary health and health status [11, 26–30]. However, the study results did not provide insight into the underpinnings of the healthcare experiences of African immigrants in the U.S. In addition, there have been a few projects aimed at understanding African immigrant health in other countries. Those studies revealed a number of barriers to African immigrants’ healthcare access that were similar to U.S. studies [31–33]. For instance, African immigrants in other countries, like in the U.S., were found to be often viewed as a part of a larger homogeneous population [34–36]. However, unlike in the U.S., in some other countries, African immigrants are grouped together in the same immigrant population with Asian and Latin Americans [37, 38]. While these studies have contributed to the African immigrant health knowledge-base, healthcare experiences and needs of Africans in other countries or continents cannot be assumed to match with those of U.S. African immigrants. For instance, the ways health insurances are operated in other countries may not be obtainable in the U.S. [39, 40]. In addition, policies that may affect health and healthcare access vary across countries [41, 42]. The current scoping review assesses existing data on the healthcare experiences and access barriers specifically of African immigrants in the U.S., with the aim of understanding the both impact of those experiences, and the putative underlying causes.

## Methods

A Scoping Review is ideal for the initial step in understanding African immigrant health due to its usefulness for exploring an issue that has not been well studied [43, 44]. Following Arksey and O’Malley’s Scoping Review framework, the review was carried out in five stages: (a) research question identification, (b) identification of relevant studies, (c) screening of studies, (d) data extraction and synthesis, and (e) presentation of results [43]. This study was approved by the Indiana University Institutional Review Board (IRB) as a part of an overarching study that examined the healthcare experiences of African immigrants.

### Identification of research questions

The specific questions this scoping review attempted to answer are: [1] What do we currently know about the healthcare experiences and needs of African immigrants in the U.S.? [2] What are the knowledge-gaps to guide the development of subsequent inquiries about African immigrant health in the U.S.?

### Identification of relevant studies

Table 1 contains a full list of the inclusion criteria. The year 1980 was chosen as the starting point for included articles because it coincided with the increased influx of African immigrants due to favorable modifications to the U.S. immigration laws [45]. The included articles were peer-reviewed, written in the English language, with research focus on the healthcare experiences of African immigrants in the U.S.. We excluded articles derived from secondary data, that is, data gathered by researchers for other purposes [46] not primarily aimed at African immigrant healthcare experiences. These data lacked the necessary variables required to examine the issue in question. Studies focused on African immigrant refugees were equally excluded, due to the unique migratory experiences of those types of subjects. Contrary to voluntary immigrants (i.e., those who decide to migrate to other countries), many refugees often flee their home countries in a hurry due to political unrest or natural disasters, with no time to prepare for their usually unpredictable journeys, which expose them to different health risks and experiences [47]. Also, many research reports have not differentiated refugees from immigrants, thereby presenting their health experiences as synonymous with voluntary immigrants [48].

**Table 1:** Inclusion Criteria for Article Selection

Criterion	Inclusion
Time period	1980–2016
Language	English
Type of article	Peer-reviewed
Population	Non-refugee African immigrants in the United States
Study focus	Healthcare experiences, needs, or health behaviors of African immigrants in the United States
Data type	Primary data collected directly from participants

Four databases were utilized in the search for relevant studies, namely: Academic Search Premier (EBSCOhost) Public Health (ProQuest), PubMed, and Google Scholar. The database searches were run from April 2016 to August 2016.^1^ In-line with scoping review recommendations [49], we first conducted a limited search of Google Scholar and ProQuest Public Health, and identified the following keywords: african immigrants, african immigration, african emigrants, african emigration, healthcare experiences, and immigrant healthcare. Following the Cochrane Effective Practice and Organisation of Care (EPOC) Database Syntax Guide [50], we used the keywords to search the included databases. This involved searching for two concepts (African immigrants AND healthcare experiences) in the subject-headings field in each study record to identify relevant studies. In PubMed, we searched the “PubMed Advanced Search Builder,” and in ProQuest Public Health Database, the Advanced Search fields. In EBSCOhost Academic Premier and CINAHL databases, we searched the Advanced Search fields in the Boolean/Phrase search modes. The first author and a health science librarian performed the article screening in duplicate through the titles first, then abstracts approach [51], and using Microsoft Office Excel spreadsheet [52]. Beyond the screening of the title and abstract for inclusion evidence, the reviewers read the full text to be certain of the article’s eligibility. The articles were divided between the two reviewers and to avoid bias, both parties were blind to each other’s decisions until completion of assigned articles. Screening discrepancies were resolved by the reviewers by meeting, comparing and discussing perspectives, and arriving at a consensus. In addition to searching the reference lists of the selected studies, we searched the reference lists of the articles identified in Google Scholar, and added the selected articles to the ones identified from the other four databases.

### Data extraction and synthesis

The articles retrieved from the different databases were stored in EndNote [53]. We followed an indicative coding approach where themes were identified from the article results [54, 55]. Using an open coding approach [56], the first author and a health science librarian exhaustively read the selected articles line-by-line, with attention to cultural meanings, then met regularly to discuss, compare, and contrast identified themes. Combining the two sets of themes, axial codes were formed, which were transformed into higher-level codes and refined with each the reading of each article, until no new themes were emerging. This process was also applicable to the selected articles which were quantitative studies, because they included qualitative data analyses with emerging themes as well. This approach to theme identification was necessary because it allows themes to emerge organically, while also keeping in check confirmation bias or assumptions [55, 57].

### Data presentation

Findings from the selected studies were synthesized into a narrative format. This form of result presentation allows a deeper insight into people’s views of themselves, especially when their experiences traverse different cultural nuances such as language and ethnicity [58, 59]. The results were presented from the perspective of the study participants regarding how the issues discussed affected them.

## Results

**Fig. 1:**
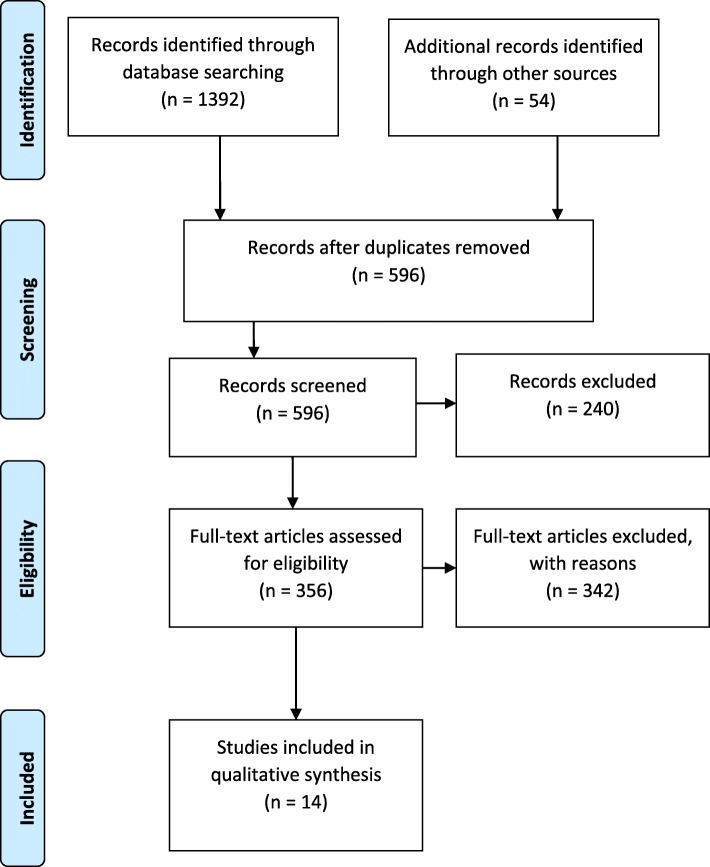
PRISMA [60] Flow Diagram of Data Search and Results

A PRISMA flowchart delineating the article screening process is represented in Fig. 1. The entire screening process yielded a total of 1446 articles, of which 206 were duplicates. Additional 644 were rejected for not meeting the inclusion criteria, including those in different languages from English, and studies not conducted in the U.S.. Of the remaining articles 596 articles, 240 were excluded for not meeting additional criteria for inclusion, including articles based on secondary data. Articles derived from secondary databases were excluded because those data were not primarily collected to examine African immigrant healthcare experiences. As such, they lacked the necessary indicators for understanding the problem. Also, studies deemed insufficiently empirical, including those conducted with surveys, but lacking qualitative data analyses, were excluded. Of the 356 remaining articles, 342 were rejected for ineligibility for inclusion, including those that had refugees as participants, studies not focused on the healthcare experiences or behavior of African immigrants, and articles we could not access.

### Description of identified studies

**Fig. 2:**
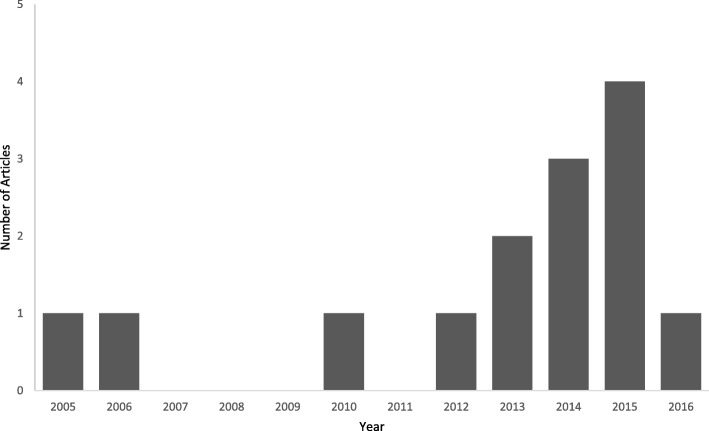
Included Articles by Year (1980–2016)

Figure 2 is a bar-chart depiction of the 14 articles that met the inclusion criteria. The horizontal and vertical lines denote the years the articles were published, and how many articles were included from each year, respectively. No article before 2005 met the inclusion criteria and no relevant articles were identified from 2007 to 2009, and in 2011. The highest number of relevant articles [4] were from 2015.

### Characteristics of included studies

A summary of the characteristics of the 14 included articles is presented in Table 2. Seven articles focused specifically on female participants, while one article concentrated on youth and the elderly. Of the other six articles, one looked at individuals over 40 years old and five focused on the general population of African immigrants.

**Table 2:** Characteristics of Included Articles

Author(s)	Year	Location	Study Design	Study Purpose	Participants
Adekeye et al. [61]	2014	Greensboro, NC	Qualitative; Photovoice; Community-based participatory research (CBPR)	Comprehend African immigrants’ views on their health and well-being, as well as barriers to their healthcare access.	Youth: 5 females and 5 males; Elderly: 1 woman and 4 men; Average age: N/A; Countries: N/A (West, North, East, South Africa)
Asare & Sharma [62]	2012	Cincinnati, OH	Quantitative; Cross-sectional	Understanding sexual communication behaviors among African immigrants, using health belief model (HBM) and acculturation.	Males: 249; Females: 163; Average age: 36.9; Countries: Ghana, Nigeria, Senegal, Cameroon, Kenya, Other
Blanas et al. [63]	2015	New York, NY	Qualitative; Focus Groups	Assess factors that affect the access to medical care of African immigrants from French-speaking countries.	Females: 12; Males: 27; Average age: 39; Countries: Burkina Faso, Guinea, Mali, Senegal
Chu & Akinsulure-Smith [64]	2016	New York, NY	Qualitative; Focus Groups & Questionnaires	Examine the health beliefs of African immigrants regarding female genital cutting (FGC), across different demographics.	Females; Average age: 35.2; Countries: Sierra Leone, Guinea, Mali, Gambia
Daramola & Scisney-Matlock [65]	2014	Detroit, MI	Quantitative; Cross-sectional (Correlational Surveys)	Examine the interaction between migration and health behaviors of African immigrant women.	Females; Average age: 56.5; Countries: Nigeria
De Jesus et al. [66]	2015	Washington, DC	Qualitative; Semi-structured Questionnaire	Explore health behaviors of East African immigrant women regarding HIV testing services.	Females; Average age: 31; Countries: Ethiopia, Eritrea, Kenya, Tanzania, Uganda
Foley [67]	2005	Philadelphia, PA	Qualitative; Focus Groups	Understand the cultural and structural barriers that affect African immigrant women’s access to HIV services.	Females; Average age: 32; Countries: Liberia, Sierra Leone, Mali, Senegal, Guinea, Ivory Coast, and Burkina Faso
Kaplan, Ahmed, & Musah [68]	2015	Kaplan, Ahmed, & Musah	Qualitative; Focus Groups	Comprehend how Ghanaian immigrants perceive their health experiences.	Females: 16; Males: 37; Average age: 45; Countries: Ghana, Gambia, Nigeria, Cameroon
Ndukwe, Williams, & Sheppard [69]	2013	Washington, DC	Qualitative; Focus Groups & Questionnaires	Assess the health behavior of African immigrants regarding breast and cervical cancer prevention services.	Females; Average age: 46; Countries: Ghana, Nigeria, Cameroon, Zambia, Ivory Coast
Raymond et al. [70]	2014	Minneapolis, MN	Qualitative; Focus Groups	Assess the health behavior and attitudes of Somali immigrant women regarding cancer prevention services.	Females; Average age: ~ 40+; Countries: Somalia
Sellers, Ward, & Pate [71]	2006	Madison, WI	Qualitative; Focus Groups	Understand the health and well-being of black African immigrant women.	Females; Average age: 44; Countries: Ghana, Cameroon, Nigeria
Turk, Fapohunda, &Zucha [11]	2015	Western Pennsylvania, PA	Qualitative; Photovoice	Assess the influence of cultural beliefs of Nigerian immigrants on healthy eating and physical activity	Females; Average age: 34; Countries: Nigeria
Vaughn & Holloway [72]	2010	Cincinnati, OH	Qualitative; Narrative Interviews	Learn from West African immigrant families in Cincinnati about their perceptions, barriers	Females: 5; Males: 5; Average Age: N/A; Countries: Mauritania, Senegal

Table 3 contains the themes and sub-themes generated from the analysis of the included studies. The theme of cultural influence comprises sub-themes including traditional beliefs and stigma-based perceptions of health, and the theme of the U.S. healthcare system was made up of sub-themes including provider attitudes and distrust of the system.

**Table 3:** Themes and subthemes generated from the analysis of included studies

Themes	Sub-themes	Examples
• Cultural Influence • U.S. Healthcare System	• Traditional Beliefs • Religiosity and Spirituality • Stigma in the community • Linguistic discordance • Cultural competence • Complex U.S. healthcare system • Cost of healthcare • Biased/hostile provider attitudes • Lack of trust of the U.S. health system	• “Why pay to find out that nothing is wrong? And why pay to find out that I have a costly problem that I can’t feel, like diabetes and high blood pressure?” • “God makes people differently and God creates people with imperfections. If you go to the doctor, God gives the doctor power to help.” • “In the eyes of a family with a person with hepatitis B, hepatitis B equals AIDS. If a family member is sick, the family no longer has the same image in the community.” • ‘If you don’t speak English, they just ignore you, or you can’t even understand your name when they call it.” • “If I’m seeing a doctor here, the doctor doesn’t understand what I’m eating in terms of the African dishes …” • “Whenever I have a hospital visit coming up, I always pray and fast for days to ensure it goes well.” • “Hospital visits are expensive; unfortunately, there are very few ethno-medical centers. In America, I don’t have access to local herbs...local herbs work!” • “If you go to a hospital and you are wearing African clothing, they don’t even want to touch you. They think we bring diseases from Africa.” • “Cancer will kill you anyway … it is a cover-up meant to use African immigrants as guinea pigs.”

### Identified themes

The two over-arching themes derived from the data analysis were the influence of culture on the provision of health care and negative experiences of the African immigrants with the U.S. healthcare system. Each one, along with the sub-themes, is discussed below.

### Cultural influence

#### Traditional beliefs

Cultural perspectives of diseases and illness determine healthcare behaviors, which in turn shape healthcare experiences. Eight articles discussed the impact of culture on the healthcare experiences of African immigrants in the U.S. [11, 61, 68, 69, 71–74]. In African immigrant communities, for example, diseases such as HIV and cancer are viewed as the result of spiritual issues? Therefore, it is not uncommon for many African immigrants to consult oracles and traditional healers in their home countries, regarding those types of health problems [72, 73]. The study by Kaplan et al., showed it was common for participants to delay office visits until the diseases or illnesses were certifiably irreversible or severe enough to halt daily activities [68]. Results from another study showed HIV-positive African immigrant women sought treatment when the condition was already in its late stage [74]. In one study, participants believed that unnecessary physician contact would lead to unwanted diagnoses. In which case an unwarranted exam would be tantamount to tempting fate. Thus, seeing a doctor was reserved for cases requiring immediate medical treatment [69]. Prior to their migration to the U.S., many African immigrants utilized herbal remedies for different health problems. In the U.S., the fear that such options may not be explored by healthcare providers, resulted in office visits and routine checks being viewed by some participants as waste of time and resources, especially given the high cost of healthcare [71]. Participants’ deference to their culture also had dietary implications. Turk and co. (2015) discovered some participants had problems with their providers’ dietary recommendations. These participants found it difficult to replace their long-held cultural perspectives regarding food and health due their contrasting outlook on body size. Whereas a big body size was considered unhealthy in the U.S., it was regarded as a sign of healthy eating in their cultures [11]. In addition, they described the fast-food culture in the U.S. not only as inescapable but problematic, due to its incongruence with their own cultural views of food preparation and consumption [61, 68].

#### Religiosity and spirituality

The influence of African immigrants’ religious and spiritual outlooks on health and well-being was presented in eight articles [61, 65, 66, 69, 70, 72, 73, 75]. In Vaughn and Holloway’s study, both the Muslim and Christian participants ascribed health status and outcomes primarily to God. They believed in spite of their efforts, their ultimate health outcomes were beyond their, or anyone’s control. Therefore, even if physicians were able to treat them successfully, that could only happen through divine assistance. Participants explained health imperfections such as illnesses and diseases as the consequences of human inadequacies, from which no one was exempt [72]. One study found that Muslim participants believed death by disease was a result of the expiration of a person’s time on earth. According to the participants, if it was God’s will that one would die from cancer for example, then there was nothing anyone could do about it. Conversely, if it was not destined for one to die yet, then despite such a disease, one would still live [66, 70, 73]. Findings from a study of key-informant focus groups indicated the African immigrant women participants were reluctant to go for cancer screening, due to their belief that their health was determined by God, who would shield them from diseases not meant for them [69]. Other participants felt Western medical care was mainly dependent on human abilities to rectify health problems, and almost negligent of the roles of spirituality and God in shaping human health [75].

This connection between spirituality and health also influenced how the participants viewed preventive healthcare. Some participants refused to answer hypothetical questions about what they would do, were they to be diagnosed with diseases such as cancer. Their rationale was that words and thoughts could affect one’s life outcomes, such as health experiences. Therefore, speaking about adverse events hypothetically was equivalent to invoking them into one’s life [65]. The Christian participants in the study expressly rejected the question, stating that it was not their lot to suffer from such diseases [69]. In addition, Adekeye et al., found a connection between African immigrants’ religiosity and dealing with mental health. Both the Christian and Muslim participants described their church and mosque attendance respectively, as necessary for coping with life’s challenges. In their views, religiosity was instrumental in shaping healthy spiritual lives, which was crucial for overall health and well-being [61].

#### Stigma in the African community

The significance of culturally-situated stigma in the healthcare experiences of African immigrants in the U.S., was identified in eight studies [62, 63, 66–71]. Blanas et al., found that one of the reasons why the African immigrant participants in their study did not make use of certain healthcare services in the U.S., was the resultant negative reactions individuals within their communities. They explained that even when the services were preventive and did not involve subjects commonly regarded as taboo, such as sexual health, they still attracted stigma from their communities [63]. This experience was applicable to participants in another study which focused on utilization of human immunodeficiency virus (HIV) prevention services. The participants emphatically preferred not knowing their status to the potential stigma and social consequences from utilizing such services, especially with HIV-positive results. According to these participants, merely going to get tested was sufficient to elicit stigma in their communities; many would deem that a positive confirmation [66]. Healthcare-related stigma within African immigrant communities is not restricted to sexually transmitted diseases or infections. Participants in the study by Ndukwe et al., explained that cancer was perceived as a curse in their communities. Consequently, the notion that the person with a cancer diagnosis has been cursed spiritually, translated to avoidance of, or cautious interaction with, the affected person [69]. Another group of participants interviewed by Raymond et al., equated cancer with HIV, in terms of perception. According to them, because both diseases were viewed by community members as death sentences and shameful, family and friends tended to be distant from the sufferer [70].

Although not linked with death as are HIV and cancer, depression is also stigmatized within African immigrant communities. Results from Sellers et al., revealed that even when participants were aware of depression, the fear of the stigma attached to being identified as depressed within their community often overrode the desire to seek treatment. According to the participants, depression was viewed as a conception and condition of white people in their communities [71]. Also, because depression was not a recognized mental health condition in many African cultures, some participants could not differentiate between health issues referred to in the U.S. as mental health problems, such as bipolar disorder, from those commonly known as “madness” in their home countries, which described mentally ill individuals roaming the streets [71]. Furthermore, the impact of health-related culturally-situated stigma within African immigrant communities, also extended beyond the affected individuals. With a cultural emphasis on a good reputation, many participants expressed fear of what would happen to their families’ standings, were it to be known that they suffered from dreaded health problems, such as cancer and mental illness. Thus, they would rather not find out their health status [66]. Even when they decided to utilize healthcare services, participants’ perceptions of privacy were an obstacle. For instance, results from Foley’s study indicated participants saw confidentiality, as managed by U.S. providers as inadequate, due to concerns about insufficient anonymity [67]. In addition, some participants suspected certain healthcare facilities were more interested in testing them unnecessarily during office visits. They feared it was only a matter of time before their private data were compromised and their livelihoods jeopardized, especially in cases of positive results for stigmatized diseases [68].

#### Linguistic discordance

Three studies discussed how the impact of language on the healthcare experiences of African immigrants in the U.S. [67, 68, 70]. Some participants experienced difficulties with translating their health needs to terms and concepts understood by U.S. providers, especially in dire circumstances. Other participants believed the language barriers they encountered were exacerbated by negative provider attitudes towards them [67]. This challenge was also pointed out by participants in the study by Kaplan et al., who felt their communication with U.S. providers would be greatly improved if the providers were more patience and less dismissive. To the participants, the poor attitudes resulted from those providers’ prejudiced expectations of language barriers from their interactions with their African immigrant patients [68].

According to some participants, productive interactions with U.S. providers entailed more than linguistic competency or availability of translators. Cultural know-how, in their views, was an inseparable aspect of effective healthcare communication. These participants’ interactions with providers were compounded by different cultural names and descriptions which were difficult to fully translate into the English language [70]. This was true even for Somali immigrants, who, despite having the highest number of translators in the public service sphere, continue to find their interactions with U.S. challenging., Participants regarded this wearisome communication with providers as a deterrent to their healthcare access, due to their fears their health needs would be unmet, or they would receive wrong treatments [70].

### Adverse experiences with the U.S. healthcare system

#### Lack of culturally-competent providers

The absence of healthcare sensitive to the backgrounds of African immigrant patients, was a pervasive theme in six articles [11, 61, 68, 70–72]. Participants in one study were disinclined towards office visits, because they feared they would result in complications, due to providers’ lack of understanding of their health needs. Not only did the participants regard those unproductive office visits as a waste of scarce resources, they considered them justifications of their lack of trust in the health system [61]. Also, participants explained that their unique cultural and healthcare backgrounds received little to no attention from the providers. In their views, those encounters constituted missed opportunities for the providers to gain broader, deeper understanding of their patients, which would have resulted in more informed interactions, and effective treatment decisions. According to the participants, even when they initiated efforts to bridge the providers’ knowledge-gap, the outcomes were still the same, due to the providers’ unaccommodating outlooks [61].

In another study, participants expressed their dissatisfaction about not being asked about their cultural or religious beliefs, which could interfere with their care. They described those omitted questions as crucial, with the power to improve effectiveness of care, though they might have seemed trivial to the providers [68]. According to other participants, the Christian or Muslim faith came with certain considerations in healthcare, including gender-roles in patient-provider interactions, and treatment options, none of which was addressed in their interactions with providers [70]. Some participants described this experience as a double-edged barrier, because their backgrounds and preferences were often excluded from their healthcare, leaving them only with the providers’ approaches, to which they had difficulties comprehending and adhering [72]. For example, some participants described how providers would often recommend difficult modifications to their diets, such as substituting one of their culturally staple foods, but without any guidance to help them achieve those goals [11]. Results from the study by Sellers et al., showed participants preferred physicians from comparable ethnicities, or, with whom they could relate. They described interactions with these types of providers as more emotionally soothing, effective, and less resource-consuming, due to quicker resolution of their health challenges [71].

#### Complex U.S. healthcare system

Difficulty navigating the U.S. healthcare system was discussed by four studies [61, 67, 71, 72]. However, this barrier did not present itself similarly in every setting. Results from the study by Adekeye et al., indicated participants ascribed their challenges with navigating the complicated U.S. healthcare system, mainly to linguistic discordance. To them, this barrier was not only in reference to low English language proficiency, but also lack of understanding of the culturally divergent aspects of the U.S. healthcare system, including terms and policies. Some participants highlighted inundation with excessive information and paperwork, with little or no guidance towards grasping their import, as a key challenge [61]. Other participants, although they had health insurance coverage, described as difficult, utilizing healthcare services, due to the challenges with deciphering which services they were eligible for and which providers were suitable [67]. Results from the study by Sellers et al. showed participants viewed the U.S. healthcare system as the most challenging aspect of their immigrant experiences. They described their healthcare experiences as replete with emotional and mental anguish, emanating from caring for the sick individual while navigating the myriad barriers to care, and knowing that the problems might not be adequately addressed [71].

#### Cost of healthcare

Five articles described the relationship between the cost of healthcare in the U.S. and the healthcare experiences of African immigrants [61, 63, 67–69]. In the study by Adekeye et al., participants identified high cost of care and the lack of western treatment alternatives, as the key barrier to their access to healthcare. Also in their views, the western assessment of African treatments as quack, inadvertently contributed to high cost of care, since it left them without cheaper alternatives [61]. Due to the exorbitant healthcare costs, some participants regarded preventive care or cases not perceptibly serious, as resource-wasting. Their rationale was that spending a lot of time and money to determine the presence of a health problem would be fiscally irresponsible, if it turned out nothing was wrong [68].

According to findings by Foley, when participants did go to the doctor, they felt it difficult to find out that the services they received were not well-covered by their health insurance. This did not only disincline the participants towards subsequent visits, it also further reduced their already distrustful views of providers, who they felt were not looking out for them as patients [67]. The problem of high healthcare cost was compounded for participants without health insurance. They could not afford the payments because they held jobs that neither provided health insurance nor paid high enough salary to allow the participants to afford healthcare services [63]. Also, participants cited the lack of transportation as one of the contributors to the high cost of care. Due to family members and friends always working, and unfamiliarity with, or absence of an easily accessible transit system, often the participants could not take advantage of some healthcare services, even including free screenings [69].

#### Biased/hostile provider attitudes

Five articles discussed the negative? role of discrimination in the healthcare experiences of African immigrants in the U.S. [61, 67, 68, 72, 73]. In one study, the participants believed that their accents or dressing styles often triggered unfavorable provider attitudes. They described their poor experiences including hostile, condescending staff approach, and provider dismissiveness and reliance on African cultural stereotypes [61]. In another study, participants described the perfunctory manners providers interacted with them, both in-person and on the telephone. The participants described that they and their health needs, being considered as undesirable encumbrances by providers, made then feel disrespects and humiliated, rendering the prospect of interacting with providers, difficult? [72].

Additionally, adverse provider behaviors towards African immigrants were not restricted to one race or ethnicity. According to Foley’s study results, participants’ accents, looks, names, amongst other background information, elicited some type of hostile attitudes from both white and black providers [67]. Other participants described their experiences with providers whose approaches were mainly derived from uninformed or stereotypical information about African immigrants, which were manifested in the providers’ questions or comments about their health issues. Still, other participants felt they were unreasonably subjected to certain tests as a result of the providers’ suspicions, founded on related stereotypes. For this reason, the participants felt targeted and avoided those locations as well as growing more distrustful of other providers [68]. Findings from Opoku-Dapaah’s study revealed a similar pattern. Participants avoided certain healthcare services, including cancer screenings, due to their suspicions that the services provided to African immigrants were more harmful than those received by their White counterparts [73].

#### Lack of trust of the U.S. health system

Three articles discussed African immigrants’ distrust of the U.S. health system and its healthcare implications [70, 71, 73]. According to the study by Sellers et al., participants’ lack of trust in the U.S. healthcare system, was inspired by the unwelcome ways they felt African immigrants and blacks in general were targeted in their personal and public domains [71]. Other participants did not believe U.S. providers had the best interests of African patients at heart, and even if they did, they felt those providers were equipped with adequate information or tactics. Also, some of the participants were convinced the health of some of their community members deteriorated after receiving western medical treatment. They expressed their unwillingness to fully acquiesce to the views of US providers, because they may not be aware of, or care about, the adverse effects of western medical approaches on African immigrants [71]. In addition, some participants’ distrust of the U.S. health system emanated from their negative views of the possible role of the pharmaceutical industry. They believed that the operational philosophy of the pharmaceutical industry primarily targeted the general public’s susceptibilities. For instance, it was their views that the expensive medical interventions were inventions of the pharmaceutical industry, primarily aimed at profiteering, while cancer screenings were smokescreens used to identify unwitting Africans as possible participants in pernicious Western health research [73]. Some participants in a study by Sellers et al. believed certain medications would result in previously absent health complications, or the exacerbation of existing ones. They were convinced they would be left to bear the brunt of the cost should that happen, while the drug manufacturers’ agenda of profiting from people’s health challenges would remain uninterrupted [71].

## Discussion

This study’s findings have highlighted two themes that underline some of the healthcare experiences of African immigrants in the U.S.. Culture and spirituality inform the ways African immigrants perceive their health and healthcare experiences, as shown by both the explicit and nuanced roles of traditional beliefs, stigma within the community, and language variance. The importance that African immigrants place on their culture with regard to health, seem supported by evidence of its health benefits. Studies of African immigrants in both the U.S. and Australia found a link between African-style diets and lower health risks, including obesity [76, 77]. Agyemang et al., found that well acculturated Ghanaian immigrants in the Netherlands had higher levels of cardiovascular risks [78], while another study showed higher colon cancer risk among African Americans in the U.S. than Africans in their home countries [79]. Understanding African immigrants’ health beliefs is critical due to potential discordance with U.S. providers’ views. For instance, a common African health perception equates higher weight with better nourishment, and lower weight with malnourishment or illness, a notion that is opposed to the predominant cultural view in the U.S. [77, 80]. Also, the perception of diseases among African immigrants has been found to emanate largely from pre-migration notion of diseases which associates most of them with spiritual origins, including cancer [73, 81]. In addition, the U.S. healthcare system presents some challenges to African immigrants, including the lack of culturally sensitive care, cost of care, complexity, and hostile provider attitudes that reinforce the lack of trust in the system. While the complexity and high costs associated with the U.S. healthcare system may not be unique to African immigrants [82, 83], their poor treatment by the U.S. medical institution is idiosyncratic [73, 84, 85]. This contributes to the pre-existing distrust of the U.S. healthcare system, which makes it difficult to engage them in intervention programs and research [86]. Contrary to the well-documented mistreatment of African Americans by the U.S. health system, including the Tuskegee Syphilis experiment [87], the underpinnings of the cynicism among African immigrants towards the U.S. healthcare system, have not yet been well identified [75].

Despite the insight provided by this study and other research examining the healthcare experiences of African immigrants, some knowledge-gaps still need to be addressed. We still need to understand the root-causes of the identified barriers. The healthcare impact of the consequences of the “black” grouping needs to be studied although it appears that being identified either as African immigrants or African Americans attracts distinct barriers, including provider biases and discrimination [61]. However, some participants in the selected studies were discouraged by the negative attitudes and biases from both white and black providers. The interpretation of barriers to African immigrants’ healthcare access in the U.S. has been oversimplified, with language often being identified [25, 88]. However, language barrier should not only include deficiency in English language proficiency as witnessed by the fact that the availability of translators for African immigrants does not guarantee the absence of communication barriers in their healthcare experiences [25]. Accents, the complex U.S. healthcare system, and culture-derived healthcare expectations, can still constitute barriers to African immigrants [18]. Even when providers communicated accurately the required dietary changes, the participants were at a loss regarding their implementation, because they were not provided any guidelines. Not only did those health goals go unmet, the opportunities for some critical patient education were lost [11, 70]. African immigrants encounter barriers to good nutrition in the U.S. as regards availability, affordability, and accessibility [61], so provider-recommended nutrition changes are insufficient.

Religion and culture also affect gender roles in African immigrant communities, which then can influence their healthcare experiences [89]. In order to meet this population’s healthcare needs, it would be necessary to examine how gender roles affect both their perception of healthcare, and the accurate understanding of provider recommendations or treatments in their communities. The antagonism between African immigrants and U.S. providers cannot be addressed without the examination of the views and attitudes of U.S. providers on African immigrant health experiences and needs. This study’s results indicated many African immigrants felt U.S. providers were not adequately equipped to address their health concerns. Finding the roots of this deep-lying distrust with input from U.S. providers, would be fundamental in improving the African immigrant healthcare experience.

## Conclusion

Very little is known about African immigrant health in the U.S.. Even as the number of African immigrants in the U.S. continues to climb exponentially, healthcare providers and policymakers have little information to guide their decision-making concerning this population. As far as we know, this is the first review of the healthcare experiences of African immigrants, which includes the overall assessment of their barriers to care or specific healthcare interventions. The findings from this review clarify some critical issues with African immigrants’ healthcare in the U.S. In addition to the identified gaps, they have also provided important cues for subsequent lines of inquiries necessary for building an understanding of the unique healthcare needs of African immigrants. However, the study still has some limitations. The language of the selected articles was limited to English. Articles published in other languages may have yielded additional findings. Also, the selected studies were limited to peer-reviewed journal articles, which excluded potential findings in grey literature, and other documents. Another limitation is the exclusion of broader studies that may report on the experiences of African immigrants within the context of other U.S. immigrants’ experiences. In addition, studies focused on refugees were not included in this study, and the participants of the selected studies were mostly from Sub-Saharan Africa. Therefore, the generalizability of the findings is limited.

## Data Availability

Data sharing is not applicable to this article as no datasets were generated or analyzed during the current study.

## Abbreviation

U.S: United States

## Footnotes

1 We ran new searches but did not identify any additional studies. While new studies have been published since then, they did not address questions guiding this review.
